# Avalanche precursors of failure in hierarchical fuse networks

**DOI:** 10.1038/s41598-018-30539-x

**Published:** 2018-08-14

**Authors:** Paolo Moretti, Bastien Dietemann, Nosaibeh Esfandiary, Michael Zaiser

**Affiliations:** 1 Department of Materials Science, WW8-Materials Simulation, FAU Universität Erlangen-Nürnberg, Dr.-Mack-Straße 77, 90762 Fürth, Germany; 2 School of Mechanics and Engineering, Southwest Jiaotong University, Chengdu, 610031 China

## Abstract

We study precursors of failure in hierarchical random fuse network models which can be considered as idealizations of hierarchical (bio)materials where fibrous assemblies are held together by multi-level (hierarchical) cross-links. When such structures are loaded towards failure, the patterns of precursory avalanche activity exhibit generic scale invariance: irrespective of load, precursor activity is characterized by power-law avalanche size distributions without apparent cut-off, with power-law exponents that decrease continuously with increasing load. This failure behavior and the ensuing super-rough crack morphology differ significantly from the findings in non-hierarchical structures.

## Introduction

Hierarchical materials are characterized by microstructure features that repeat on different length scales in a self-similar fashion. Biological materials provide compelling examples. Collagen, for instance, exhibits a hierarchical fiber organization which at different length scales comprises molecules, microfibrils, fibers, and fiber bundles^1^. Such complex organization was shown to provide enhanced toughness over assemblies of isolated collagen molecules. Several authors (see e.g.^2^) have suggested that hierarchical organization may delay or prevent the nucleation and spreading of critical flaws which control failure of non-hierarchical heterogeneous materials^3,4^. Models of hierarchical materials have mostly used hierarchical generalizations of the well-known equal-load-sharing fiber bundle model (ELS-FBM) which is a mean-field model for brittle fracture in disordered materials (see e.g.^5^). In hierarchical variants, fibers are recursively grouped into bundles and load is assumed to be distributed equally among the intact fibers within each bundle - a salient feature which makes such models amenable to analytical treatment as renormalization arguments can be used to deduce the overall strength^6^ and the statistics of damage accumulation. Hierarchical fiber bundle models have been used in the context of biomaterials (see e.g.^7^) and also of composites^8^. A variant which consists in envisaging the structural elements of a hierarchical fiber bundle not as simple fibers but as chains-of-bundles does not greatly alter the basic conceptual framework since, at least in the limit of elastic-brittle local constitutive behavior, the properties of a bundle can be inferred from those of the single fibers using standard methods^9^ and those of a chain-of-bundles then be deduced by weakest-link statistics. Models of this type were introduced for a speculative nanotube-space-elevator cable^10^ and for hierarchical bio-materials^7^.

Practically all investigations of hierarchical fiber bundles focus on the effective strength of the hierarchical structures, whereas fundamental questions concerning the *nature* of the failure process (critical behavior vs. sub-critical crack nucleation-and-growth) and the concomitant nature and statistics of precursor events have received little attention^9^. In fact, because of their mean-field nature, ELS-FBM and their generalizations are not well suited for investigating spatial patterns of damage accumulation and failure. In the present work we therefore depart from the fiber bundle paradigm. To explore how hierarchical organization affects the precursor activity in the run-up to failure and ultimately changes the *mode* of failure, we formulate for the first time hierarchical generalizations of the well-known random fuse network (RFN)^11,12^ which, unlike ELS-FBM, is known to capture essential features of spatial stress patterns occurring during failure of continuous media such as the *r*^−1/2^ character of crack-tip stress singularities. At the same time we emphasize that RFN models, representing a scalar caricature of tensorial elasticity, can provide a quantitative description of fracture of materials only in exceptional cases, such as tearing of thin sheets loaded in anti-plane shear or uniaxial loading of materials with zero Poisson ratio (for an example see^13^).

## Results

### Construction and statistical properties of hierarchical fuse networks

**Figure 1:**
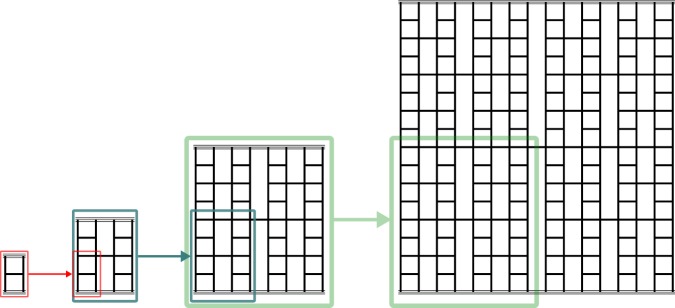
Hierarchical fuse network. Deterministic recursive construction of a hierarchical fuse network (D-HFN); a network of *n* hierarchical levels consists of 4 modules, each of which represents a network of *n* − 1 hierarchical levels; a network of *n* + 1 hierarchical levels is generated from a *n*-level network by substituting each of these 4 modules by the level-*n*-network.

Our aim is to generalize fuse network models in such a manner that they can be used as concept models for investigating the impact of hierarchical architecture on the mode of failure of materials, highlighting substantial differences between hierarchical and non-hierarchical materials, and drawing analogies with the behavior of hierarchically architectured systems outside the realm of materials mechanics. To this end, we generalize the RFN model into a hierarchically cross-linked network of breakable fibers of heterogeneously distributed strength, which we denote as Hierarchical Fuse Network (HFN). The construction of such a network is illustrated in Fig. 1. The network consists of inter-connected links of unit length and unit conductance (fuses) that are contained between two bus bars, which we visualize as located at the top and the bottom of the network. Through the bus bars, a load on the network is imposed, either in the form of a prescribed voltage between top and bottom bar or in terms of a prescribed total current flowing from top to bottom. The vertical direction is hence referred to as the loading direction (or load parallel-direction), whereas the horizontal direction is referred to as the load-perpendicular direction.

As zeroth-order module we define a vertical (load-parallel) link. The first-order network, which in Fig. 1 consists of four zeroth-order modules plus one load-perpendicular cross-link, is referred to as the HFN generator (other forms of generators are explored in the Supplementary Information). A hierarchical network is then constructed recursively as follows: From the first order network, *n* = 1, we obtain a network of order *n* = 2 by replacing each zeroth-order module with the generator itself. Hence, the second order network consists of four generator (first-order) modules plus a connecting horizontal cross-link of length 2^2^ − 1. Accordingly, a network of order *n* + 1 is constructed by replacing, in a network of order *n*, each module of order *n* − 1 by a module of order *n* and connecting the central network by a link of length 2^*n*^ − 1. A network of order *n* that has been constructed in this manner represents an anisotropic structure consisting of 2^*n*^ − 1 load-parallel wires of length 2^*n*^ which are cross-linked in a hierarchical manner. The quantity *L* = 2^*n*^, which defines the linear dimension of the network, is also referred to as the network size.

**Figure 2:**
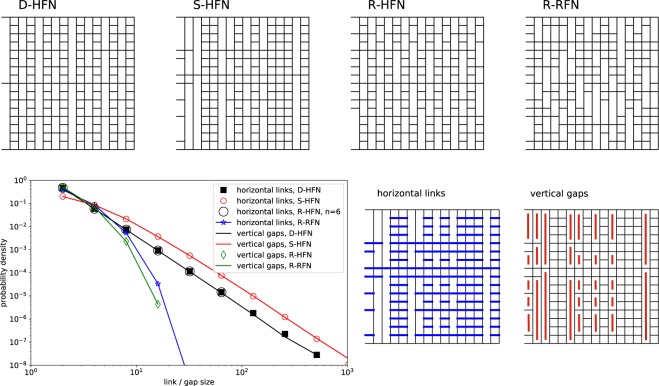
Hierarchical fuse network variants. Top: Structure of regular and randomized RFN, *n* = 4: regular D-HFN, S-RFN with random permutation of both columns and rows, R-HFN with randomly rotated rows, R-RFN reference random fuse model with randomly placed cross-links; Bottom: size statistics of load-perpendicular cross-links and load-parallel “gaps” for the different network variants, using logarithmic binning into bins of size 2^*m*^. Data in the plot is for systems with *n* = 9, except where explicitly noted. The plot is accompanied by a graphical representation of links and gaps in a S-HFN with *n* = 4 and with periodic boundary conditions along the load-perpendicular direction.

In addition to the deterministically constructed HFN (henceforth referred to as D-HFN), we consider several randomized variants. To construct these, we impose periodic boundary conditions in the load perpendicular direction on the D-HFN, by replacing the central cross-link of length *L* − 1 by one of length *L* and closing periodically. A row is defined as a set of load-perpendicular links that share the same vertical position, and a column is defined as a set of load-perpendicular links that share the same horizontal position. Variant networks as illustrated in Fig. 2, top, are then constructed as follows: (i) A network constructed by starting from a D-RFN and then first randomly reshuffling the columns and then the resulting rows is denoted as S-HFN (first reshuffling the rows and then the columns produces statistically equivalent results). (ii) A network constructed by independently rotating the rows of a D-HFN by random integers *i* ∈ [0…*L* − 1] across the periodic boundaries is denoted as R-HFN. (iii) We take the HFN cross-links and distribute them randomly over the *L*^2^ possible cross-linking sites. This process creates a non hierarchical structure with equal degree of cross-linking, which we denote as a reference random fuse network, R-RFN.

The cross-link structure of the different HFN variants can be statistically characterized in two manners illustrated in Fig. 2, bottom left. We may focus on the row structure and envisage the network as an assembly of load-perpendicular cross-links, where the length of a cross-link is understood as the number of horizontally connected elementary links. Alternatively, we may envisage the network as an assembly of load-parallel gaps, where the length of a gap is referred to as the number of vertically adjacent locations where an elementary cross-link is missing. Interestingly, the different network variants differ substantially in the statistics of these elements, see Fig. 2.

For the D-HFN, both cross-link lengths *n*_cl_ (number of horizontally connected cross-links) and gap lengths *n*_gp_ (number of vertically adjacent gaps) are power-law distributed, $p({n}_{{\rm{gp}}})\propto p({n}_{{\rm{cl}}})\propto {n}_{{\rm{gp}},{\rm{cl}}}^{-\kappa }$ where the recursive construction implies the exponent *κ* = 3. The random re-shuffling of columns and rows which produces a S-HFN does not change these power-law distributions of cross-link lengths and gap lengths. While the short-length behavior of the distributions is slightly modified, the power-law exponent of the distributions which governs the decay at large scales is unaltered (red circles and connecting red line in Fig. 2). The R-HFN possesses by construction the same cross-link statistics as the D-HFN since rotating a row across the periodic boundaries does not change the lengths of the connected cross-links. However, the gap statistics in this case becomes exponential, see Fig. 2. Finally, for the R-RFN both the cross-links and the gaps are exponentially distributed as expected when cross-links are randomly distributed over the network. We thus have three kinds of networks: Networks with power-law distributed gaps and cross-links (D-HFN and S-HFN), networks where cross-links are power-law distributed but gaps are exponentially distributed (R-HFN) and networks where both links and gaps are exponentially distributed (D-HFN).

Points where links are mutually connected are referred to as nodes; a network of size *L* has *L*(*L* − 1) nodes. Once the network morphology is established, we assign to each link a critical current: The link connecting nodes *k*, *l* fails once the current *I*_*kl*_ flowing through this link exceeds the critical value *t*_*kl*_. Stochastic material heterogeneity is mimicked by taking the thresholds *t*_*kl*_ to be independent random variables which we assume to be uniformly distributed between 0 and 1, representing an assembly of highly unreliable elements. Other critical current distributions yield qualitatively similar results, see Supplementary Information.

### Behavior under load

The networks can be loaded by adjusting the voltage difference *V* between the bus bars to maintain a fixed total current *I* (load control), or vice versa (voltage control). Except where explicitely noted, in the following we present results for the case of load control. The voltage *V*_*k*_ at node *k* represents a displacement-like variable, while the currents *I*_*kl*_ flowing between nodes represent stress-like variables. The equilibrium equations for this scalar model of elasticity result from Kirchhoff’s node law, imposing that the algebraic sum of all forces (currents) at a node must be zero. We follow the standard loading protocol for quasi-static RFN simulations^12^ (see Methods section). Under load control, the external load (the imposed current) is increased to the precise level where the first link breaks and then kept fixed while link failure leads to load re-distribution which may trigger further failures: damage accumulates through bursts of local failures (avalanches). The number of failures occurring as a consequence of internal load re-distribution at fixed total current defines the avalanche size *s*. Subsequent to an avalanche the load is again increased to induce link breaking, and this is repeated until global failure disconnects the network.

**Figure 3:**
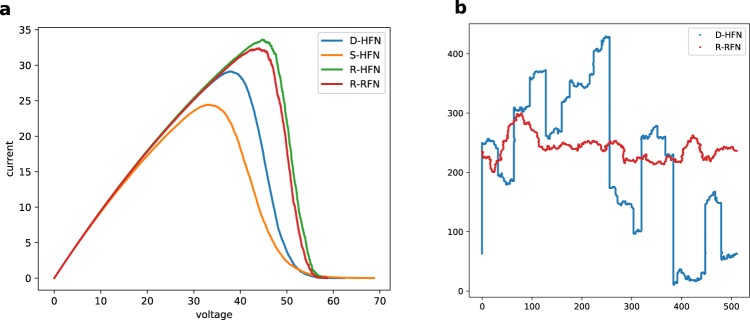
Behavior of HFN under load. (**a**) Averaged current-voltage curves (voltage control) for the different network variants, with *L* = 512; (**b**) crack shape in a D-HFN and a reference R-RFN, with *L* = 512.

Figure 3a shows average current-voltage characteristics for the HFN (voltage control). Comparison between the different simulated network variants demonstrates that R-HFNs possess the highest peak current (which corresponds to the failure current in current control), followed by the reference RFN, D-HFN and S-HFN. While the peak currents for all morphologies are of the same order of magnitude, the crack patterns are significantly different between D-HFN and S-HFN on the one hand, and R-HFN and reference RFN on the other hand. We show in Fig. 3b a typical crack profile for a D-HFN close to failure together with a crack profile for a reference RFN. The RFN crack profiles exhibit typical self-affine shapes as studied extensively in the literature on RFN models (see e.g.^14^). The crack shape in both D-HFN and S-HFN is qualitatively different. In these networks the hierarchical structure with a power law distribution of vertical gaps imposes wide discontinuous jumps in the crack profile which are visually reminiscent of crack profiles encountered e.g. in bone^15^.

### Avalanche statistics

**Figure 4:**
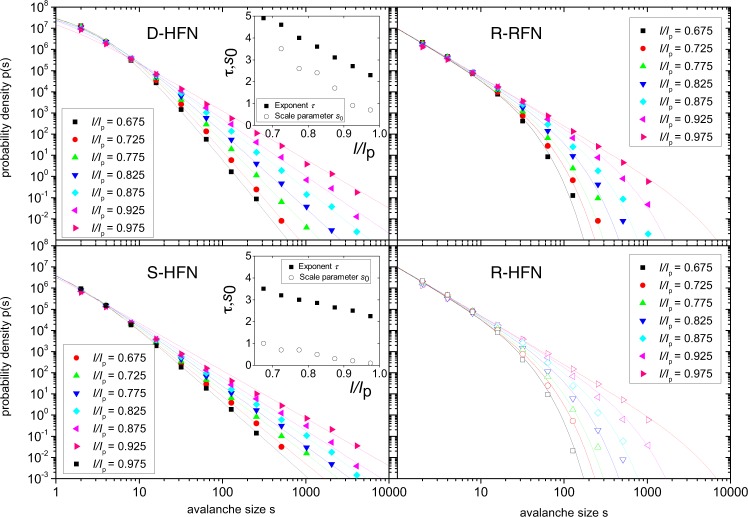
Avalanche statistics. Avalanche size distributions for HFN and random reference networks of size *L* = 512; top left: D-HFN, bottom left: S-HFN, the lines represent fits of Pareto distributions as given by Eq. (2), the fit parameters are shown in the insets; top right: R-RFN, bottom right: R-HFN, the lines represent a common fit to all data (R-RFN and R-HFN) using Eq. (1); all distributions are averaged over 3 × 10^5^ realizations of the respective networks.

To understand the differences between D-HFN and S-HFN on the one hand, and R-RFN and R-HFN on the other hand, we study the size distributions of avalanches of link breakings that occur prior to global system failure. We resolve these distributions with respect to the applied load (current): the loading curve is subdivided into load value intervals and avalanche size distributions are computed separately for each interval. For non-hierarchical RFN, the statistics of precursors to failure is well established: avalanche activity in the run-up to failure is characterized by truncated power-law distributions of avalanche sizes of the form

1 $$P(s)=N{s}^{-\tau }\exp [-\frac{s}{{s}_{0}}{(1-\frac{I}{{I}_{{\rm{p}}}})}^{1/\sigma }]$$

with a fixed exponent *τ* and a cut-off that increases with load and diverges at the point of failure^14,16^. More realistic spring or beam models^17,18^ yield similar results. The same picture can also be found in our own simulations of R-RFN where the lateral cross-links between the load carrying fibers are located randomly to create a non-hierarchical reference structure, see Fig. 4 top right, where the avalanche size distributions can be well fitted by Eq. 1 with *τ* = 2.3 and 1/*σ* = 1.95. For comparison, ref.^14^ reports values of *τ* ≈ 2 and 1/*σ* ≈ 1.4 with a weak dependence on lattice morphology. While these exponent values differ from the mean-field values *τ* = 1.5 and *σ* = 1, the avalanche size distributions are of the same type as for ELS-FBM. A similar picture emerges from simulations of R-HFN as shown in Fig. 4 bottom right. In the case of D-HFN and S-HFN, the picture is completely different as avalanche sizes are distributed as power laws with continuously varying exponents throughout the loading curve without an apparent cut-off. The distributions cannot be meaningfully fitted by Eq. 1 but are well represented by modified Pareto distributions,

2 $$P(s)=\frac{N}{{(s+{s}_{0})}^{\tau }}$$

where now the exponent *τ* decreases with increasing load *I* in an approximately linear manner (Fig. 4, left-hand side). Only at the peak current the distributions for HFN and RFN approach each other, as in the former case the cut-off diverges while for the HFN the exponent of the scale free distribution approaches the asymptotic value *τ* = 2.3 that also characterizes the random reference network. We may thus conclude that, while RFN exhibit a kind of critical-like behavior which is scale free only at the point of failure, in D-HFN and S-HFN such scale free behavior is a robust, intrinsic feature of the dynamics as the avalanche size distributions have power-law characteristics without cut-off even far away from the peak load.

### The role of the network structure

In order to understand the origin of this robust scale free behavior, we note that hierarchical modular organization has been known to produce generic scale invariant behavior in systems apparently unrelated to materials mechanics. Models of activity propagation in both real and computer generated mappings of the human brain, in particular, have produced similar avalanche size distributions with continuously varying, non-universal exponents^19^. Power-law distributed avalanche sizes are believed to be a direct consequence of the morphology of the brain networks, which are organized into a hierarchy of modules of exponentially increasing size yet exponentially decreasing number. Thus, scale free dynamic patterns are a consequence of scale invariant hierarchical organization of the underlying network, a consideration that holds for processes as varied as activity propagation and percolation, and is backed by renormalization results^20^. In such structures, critical points marking phase transitions may be replaced by extended critical-like regions (“Griffiths phases”) as discussed by Moretti and Muñoz^19^ and here for the first time observed in the context of mechanical breakdown.

**Figure 5:**
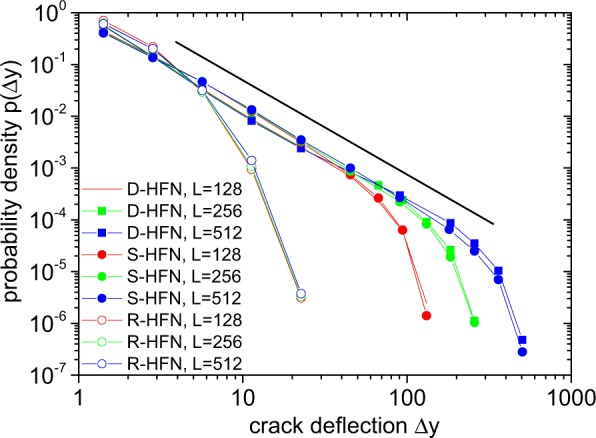
Crack morphology. Distributions *p*(Δ*y*) of crack deflections in the load-parallel direction for deterministic, shuffled and rotated HFNs of sizes *L* = 128, *L* = 256 and *L* = 512: the straight line represents a power law of exponent *θ* = 1.75.

To understand how hierarchical organization ensures the scale free statistics of precursor activity, we compare the behavior of the different network variants. The behavior of the D-HFN and the randomly re-shuffled S-HFN is essentially the same: in both cases we observe power-law avalanche size distributions with an exponent *τ* that decreases towards the value at failure, *τ* = 2.2, as an approximately linear function of the current *I*. At large avalanche sizes, the distributions are very clean power laws. At small sizes, deviations show up which can be characterized by a Pareto scale parameter *s*_0_ that goes to zero in a linear manner as the current approaches the critical value *I*_p_ (Fig. 4 left-hand side and insets). Networks with differing generators and differing threshold current statistics show similar behavior (see Supplementary Information). Differences between D-HFN and S-HFN concern only the numerical values of *τ* and *s*_0_, which are both smaller for the S-HFN but approach common values at failure. The behavior of the R-HFN is qualitatively different from the hierarchical networks but identical to that of a reference network with completely random cross-links. In both cases, one finds the same truncated power-law distributions with exponent *τ* = 2.2 and a cut-off that diverges as the current approaches *I*_p_. Since the R-HFN has the same distribution of cross-link lengths as the D-HFN but the same exponential distribution of gap sizes as the random reference network, we can safely conclude that the robust scale-free behavior of the avalanche statistics in the hierarchical networks results from the scale-free gap size distribution, both in deterministic network models (D-HFN) and in more realistic randomized variants (S-HFN). This expectation is in line with the fracture pattern of a D-HFN in Fig. 3b, which demonstrates that the final crack is deflected on all scales by the vertical gaps which interrupt stress transmission at the crack tip, leading to a super-rough crack morphology. This qualitative idea is borne out by a quantitative analysis of the distribution of vertical deflections Δ*y* of the crack which is characterized by truncated power laws, $p({\rm{\Delta }}y)\propto {\rm{\Delta }}{y}^{-\theta }\varphi ({\rm{\Delta }}y/L)$ where the cut-off is given by the system size, as shown in Fig. 5. The observed exponent *θ* = 1.75 differs from the value *θ*′ = 2 for the gap size distribution along a horizontal line, indicating non-trivial dynamics as stress concentrations at the tip of the emergent crack interact with the network morphology. R-RFN and R-HFN, on the other hand, exhibit an exponential distribution of Δ*y* with an average deflection that is slightly larger than the mean gap size.

## Discussion

We have proposed a simple model of stress redistribution and failure in a model material with a hierarchical microstructure. Analogously to heterogeneous materials that lack multi-layer hierarchical organization, damage accumulation proceeds intermittently in the form of avalanches, which are broadly distributed in size. We observe however that in the hierarchical case this phenomenology cannot be interpreted as critical behavior in the vicinity of a continuous phase transition, as paradigmatically implemented in fiber bundle models with equal load sharing. Avalanches with power-law distributions without apparent cut-off are observed *generically*, i.e. for any value of the applied load. Avalanche exponents vary continuously, suggesting that the concept of universality class cannot be invoked. We argue that failure patterns, as well as deformation/load patterns, arise naturally from the hierarchical microstructure of the deforming medium, which is scale invariant by construction. The fracture patterns reflect the same scale invariance and strongly differ from the self-affine crack morphologies generally observed in non-hierarchical random fuse networks^14^. Fracture occurs not by nucleation-and-propagation of a critical crack as typical of non-hierarchical or R-RFN (see Supplementary Video 1), but by coalescence of multiple, widely separated flaws as crack propagation is interrupted by the presence of hierarchically distributed gaps on all scales (see Supplementary Video 2). This intrinsic tendency of the hierarchical-modular microstructure to localize damage reflects the generic capability of hierarchical networks to localize activity patterns, reported for a wide range of biological^19,21,22^ and even information processing networks with hierarchical microstructure^23^. Further work is needed to systematically quantify how the scale-free dynamics of damage accumulation and the ensuing crack profiles relate to the parameters governing the scale invariant microstructures (exponents of the distribution of link and gap sizes), which can be “tuned” by changing the number of horizontal and vertical links in the D-HFN generator. This tuning capability may represent the ultimate advantage of hierarchical microstructures, as recently suggested in the context of friction^24^.

The results obtained here represent first steps towards a qualitative understanding of failure processes in hierarchically organized materials. For a quantitative description of failure in complex biomaterials, which combine hierarchical morphology with a composite microstructure containing multiple phases, it will be necessary to go beyond the simplified caricature of load transfer in terms of a scalar load variable that is inherent in fuse models and move towards models that allow for a fully tensorial description of deformation and failure of hierarchically organized multi-phase composites.

## Methods

Simulations are conducted using the standard quasi-static method for the Random Fuse Model^12^. At every iteration, both current and voltage are tuned to the lowest values which ensure that one link is broken. This is obtained as follows: (i) a fixed external *V* = 1 is applied to one of the buses, while the other is kept at *V* = 0 (ii) voltages at each node *k* are computed solving Kirchhoff’s node law, (iii) currents *I*_*kl*_ at each link *kl* are computed using Ohm’s law, (iv) the link with the maximum *I*_*kl*_/*t*_*kl*_ is removed (*t*_*kl*_ being the random threshold assigned to the link), (v) the global values of *V* and *I* are adjusted by the factor *t*_*kl*_/*I*_*kl*_ (which yields the failure of link *kl*), and are recorded in the *I* − *V* curve. The resulting quasi-static *I* − *V* curve allows one to extract information both for current- and voltage-control loading schemes. In the case of current-control, the size of an avalanche is defined as the number of links that fail without any further increase in the applied load. Avalanche statistics data, as in Fig. 4, are obtained by subdividing the interval of applied currents into sub-intervals. Avalanche size distributions are computed separately for each sub-interval.

## Electronic supplementary material

Supplementary Information
