# Supplementary material for: Avalanche precursors of failure in hierarchical fuse networks
Source: Sci Rep. 2018 Aug 14;8:12090. doi: 10.1038/s41598-018-30539-x (PMC6092438; doi:10.1038/s41598-018-30539-x)
Supplement: Supplementary file 1 — Supplementary Information [file 41598_2018_30539_MOESM1_ESM.pdf]

Supplementary Information :

Avalanche precursors of failure in hierarchical fuse networks

Paolo Moretti[1,*], Bastien Dietemann[1], Nosaibeh Esfandiary[1] and Michael Zaiser[1,2]

[1]*Dept. of Materials Science, WW8-Materials Simulation, FAU Universität Erlangen-Nürnberg, Dr.-Mack-Straße 77, 90762 Fürth, Germany*
[2]*School of Mechanics and Engineering, Southwest Jiaotong University, Chengdu 610031, China*
[*]paolo.moretti@fau.de

1 Effect of local disorder statistics

In RFN and HFN models, disorder is introduced in terms of the statistical distribution of local failure currents t. In addition to the uniform distribution on the interval $t \in [0, 1]$ considered as default in our paper, we also investigated Weibull distributed failure currents with the cumulative distribution function

$$P(t) = 1 - \exp[-(t/t_0)^{\beta}] \tag{1}$$

where the exponent β controls the degree of disorder, with small values of β corresponding to a high scatter of t. The parameter t_0 is adjusted to yield the same mean value $\langle t \rangle = 1/2$ as for the default distribution. Results are shown in Fig. 1 for S-HFN with Weibull distributed failure currents. We consider the Weibull exponent values $\beta = 1.5$ (corresponding to a standard deviation of t values that is approximately equal to the default distribution) and $\beta = 4.5$, corresponding to a much less disordered system.

The overall failure scenario is unaffected by the local threshold distribution: The avalanche size distributions are characterized by power-law statistics without an apparent cut-off. Fit by the Pareto distribution

$$P(s) = N/(s + s_0)^{\tau} \tag{2}$$

yields τ values that decrease in the approach to failure towards the value $\tau \approx 2$. While the exponents away from the critical current depend on the Weibull modulus, being larger for larger modulus, the asymptotic value of τ near failure appears to be independent of the threshold statistics.

A significant difference between the uniform default distribution and the networks with Weibull distributed thresholds is however evident at small avalanche sizes where

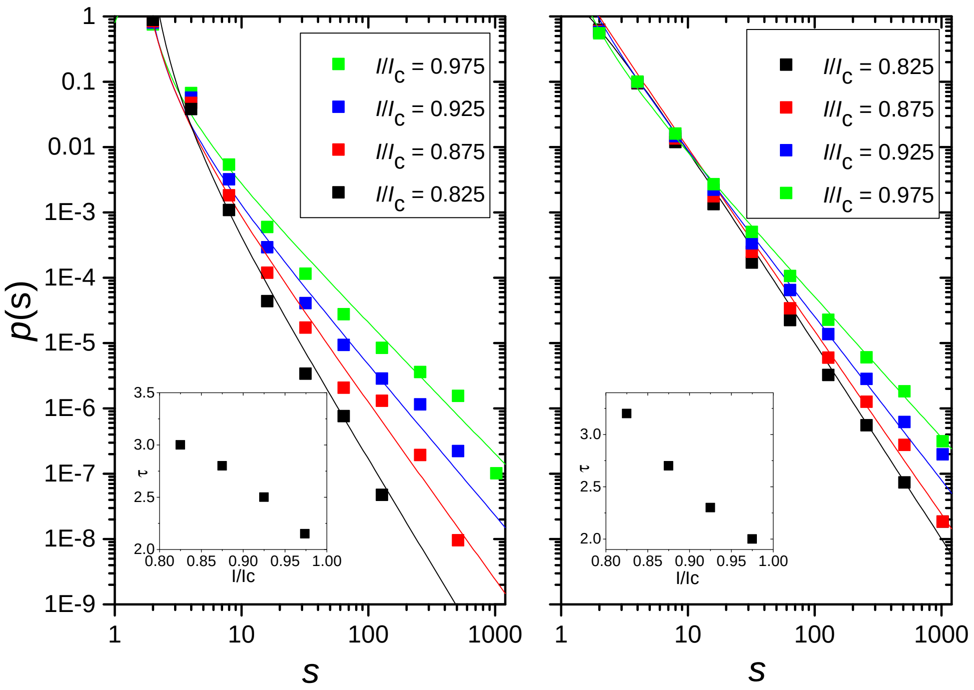

Figure 1: Avalanche distributions in the run up to failure for S-HFN with Weibull distributed local thresholds, left: Weibull exponent $\beta = 4.5$, right: Weibull exponent $\beta = 4.5$; the insets show the evolution of the avalanche exponent τ as a function of the imposed current; the lines represent fitted Pareto distributions.

Weibull distributed failure currents lead to a positive curvature of the double-logarithmic $P(s)$ plot, corresponding to negative values of the fit parameter s_0. For the default uniform distribution, by contrast, the curvature is negative and s_0 has a positive value.

2 Influence of the morphology of the hierarchical network.

The morphology of the network can be "tuned" by changing the elementary unit ("generator") at level $n = 1$ of the hierarchical construction. As in the default structure, in each iterative step a network of $n+1$ hierarchical levels is generated from a network with n hierarchical levels by replacing each module of order $n-1$ with the order-n-structure. The statistics of cross-link and gap lengths then depends on the numbers of horizontal cross links and vertical links within the generator.

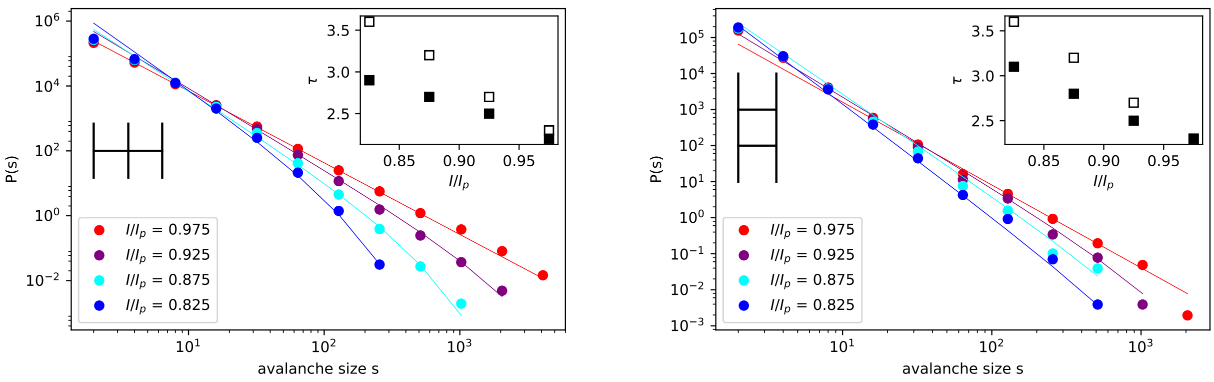

Figure 2: Avalanche distributions in the run up to failure for D-HFN with two different generator variants as shown in the graphs; the insets shows the evolution of the avalanche exponent τ as a function of the imposed current (full squares) in comparison with the values for a D-HFN with the default generator considered in the main paper (open squares).

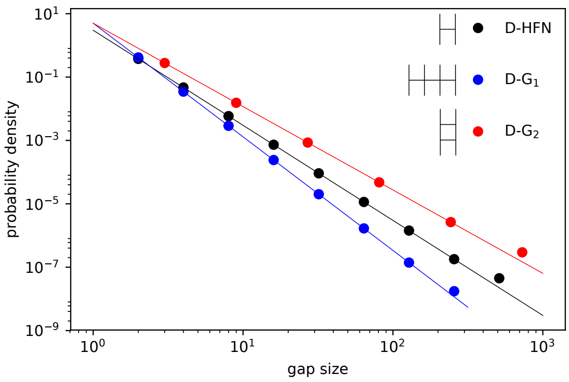

Figure 3: Distribution of load-parallel gap sizes for the three different generators studied. Black: Default generator considered in the main paper. Red and blue: generators G1 and G2 as illustrated in Fig. 2. The full lines represent power laws with the theoretical exponents $k = 3$ (default generator), $k = 2 + \log_2 3 \approx 3.58$ (generator G1) and $k = 2 + \log_3 2 \approx 2.53$ (generator G2).

Fig. 2 shows avalanche size distributions for two generator variants. After generating the structures, thresholds were assigned to all links by assuming the default uniform distribution. As for the standard generator considered in the main paper, the double-logarithmic $P(s)$ curves exhibit positive curvature at small s and cross over to non-truncated power laws at large s, a behavior that can be well fitted by modified Pareto distributions as given by Eq. (2). Again, the avalanche exponent τ increases with increasing imposed current towards the value $\tau \approx 2$ which marks the failure current. The absolute values of τ away from the failure current are different for the different structures but there is no straightforward correlation with parameters such as the exponent of the vertical gap distribution (see Fig. 2): both variant generators G1 and G2 exhibit smaller τ values for the same current than the default structure, even though the gap exponents deviate in opposite directions. This indicates that more work may be needed

to understand the connection between morphology and avalanche statistics within the HFN paradigm.

3 Cumulative avalanche distribution and size of the final avalanche

In RFN models, failure has critical-like characteristics in the sense that, under current control, the current-dependent avalanche size distribution exhibits the scaling form

$$P(I, s) = N s^{-\tau} \exp\left[-\frac{s}{s_0}\left(1 - \frac{I}{I_\mathrm{p}}\right)^{1/\sigma}\right]. \tag{3}$$

A corollary to this scaling behavior is that the cumulative distribution $P(s) = \int P(I, s) dI$ is a straight power law with exponent $\tau + \sigma$. The final avalanche which occurs at the peak current and disconnects the network is an outlier to that distribution, see [1].

In the HFN case we have a slightly different picture. The superposition of distributions with different exponents does not lead to a straight power law but, on a log-log plot, to a convex curve. The slope of this curve continuously decrases and, in the regime of large avalanches, is controlled by the value of τ near the peak current Fig. 4. However, the behavior is similar to the RFN case in the sense that the final avalanche still represents an outlier with different statistics from those of the pre-peak avalanche sequence.

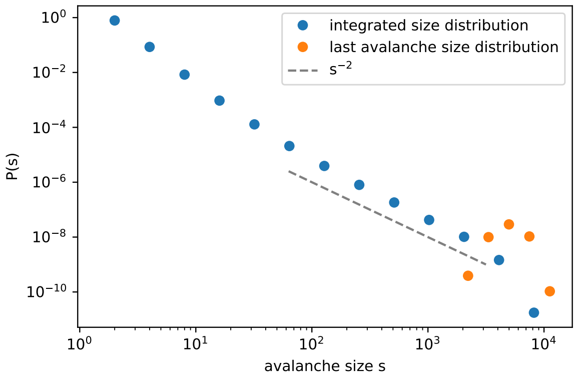

Figure 4: Cumulative distribution of avalanche sizes; values are given separately for avalanches occurring before the peak current and for the final avalanche.

4 Supplementary videos

Supplementary videos show the evolution of current profile and crack morphology under conditions of increasing voltage, for a non-hierarchical and a hierarchical system respectively. System sizes are $L = 512$ ($n = 9$). In the current profile snapshots, we also mark (as black dots) the broken links that will contribute to the final crack profile, as soon as they are broken.

Supplementary Video 1

Left: Current profile and crack morphology for a non-hierarchical system (R-RFN). Fracture occurs by nucleation-and-propagation of a critical crack. Brighter colors indicate higher currents. Right: location in the I-V curve of the configuration depicted on the left, in a voltage-controlled simulation.

Supplementary Video 2

Left: Current profile and crack morphology for a hierarchical system (D-HFN). Fracture occurs by coalescence of multiple, widely separated flaws, as crack propagation is interrupted by the presence of hierarchically distributed gaps on all scales. Brighter colors indicate higher currents. Right: location in the I-V curve of the configuration depicted on the left, in a voltage-controlled simulation.

References

[1] Zapperi, S., Nukala, P. K. V. V. and Simunović S. Crack roughness and avalanche precursors in the random fuse model. *Phys. Rev. E* **71**, 026106 (2005).
